# Deep learning-based clustering approaches for bioinformatics

**DOI:** 10.1093/bib/bbz170

**Published:** 2020-02-01

**Authors:** Md Rezaul Karim, Oya Beyan, Achille Zappa, Ivan G Costa, Dietrich Rebholz-Schuhmann, Michael Cochez, Stefan Decker

**Affiliations:** 1 Fraunhofer Institute for Applied Information Technology FIT, Schloss Birlinghoven, Sankt Augustin, Germany; 2 Information Systems and Databases, RWTH Aachen University, Aachen, Germany; 3 Insight Centre for Data Analytics, National University of Ireland Galway, Ireland; 4 Institute for Computational Genomics, RWTH Aachen University Medical School, Aachen, Germany; 5 German National Library of Medicine, University of Cologne, Germany; 6 Department of Computer Science, Vrije Univeriteit Amsterdam, The Netherlands

## Abstract

Clustering is central to many data-driven bioinformatics research and serves a powerful computational method. In particular, clustering helps at analyzing unstructured and high-dimensional data in the form of sequences, expressions, texts and images. Further, clustering is used to gain insights into biological processes in the genomics level, e.g. clustering of gene expressions provides insights on the natural structure inherent in the data, understanding gene functions, cellular processes, subtypes of cells and understanding gene regulations. Subsequently, clustering approaches, including hierarchical, centroid-based, distribution-based, density-based and self-organizing maps, have long been studied and used in classical machine learning settings. In contrast, deep learning (DL)-based representation and feature learning for clustering have not been reviewed and employed extensively. Since the quality of clustering is not only dependent on the distribution of data points but also on the learned representation, deep neural networks can be effective means to transform mappings from a high-dimensional data space into a lower-dimensional feature space, leading to improved clustering results. In this paper, we review state-of-the-art DL-based approaches for cluster analysis that are based on representation learning, which we hope to be useful, particularly for bioinformatics research. Further, we explore in detail the training procedures of DL-based clustering algorithms, point out different clustering quality metrics and evaluate several DL-based approaches on three bioinformatics use cases, including bioimaging, cancer genomics and biomedical text mining. We believe this review and the evaluation results will provide valuable insights and serve a starting point for researchers wanting to apply DL-based unsupervised methods to solve emerging bioinformatics research problems.

## 1 Introduction

Clustering is a fundamental unsupervised learning task commonly applied in exploratory data mining, image analysis, information retrieval, data compression, pattern recognition, text clustering and bioinformatics [1]. The primary goal of clustering is the grouping of data into clusters based on similarity, density, intervals or particular statistical distribution measures of the data space [1–3], e.g. clustering gene expressions (GEs) can reveal groups of functionally related genes in which genes with a small distance share the same expression patterns and might be similar [4]. Such analysis determines which genes are switched ‘on’ or ‘off’ under certain conditions [4–6]. Another example is the clustering of genes or biomedical images by learning the hidden patterns from an unlabeled dataset [7]. Further, visualizing, interpreting and analyzing high-dimensional and large-scale biological data in its unstructured entirety can be perplexing unless the data is organized into clusters.

Clustering based on biological entities such as genes, diseases, proteins, pathways and small molecules depends on the amount, quality and type of input data [8] or samples (e.g. patients or distinct cells). Although a vast amount of biological data are being generated from numerous ubiquitous medical devices, applications of clustering still limited within genomic medicine and microarray analysis focusing on gene clustering with a limited amount of data [9, 10]. Accordingly, cluster analysis covering bioimaging, human-genetics, plant and animal ecology, biomedical texts and genomic data are not fully explored and still in their infancy when compared to what has been investigated for microarrays [11], e.g. RNA-Seq has become the de-facto technology for GE-level measurements and offers several advantages over microarrays. Further, single-cell experiments have become an emerging bioinformatics research topic, where clustering is a crucial part of the analysis [12].

However, since cluster analysis itself is not one specific algorithm, various techniques can be applied and differ in terms of understanding of what constitutes a cluster and how to efficiently find them. In practice, different problems require different similarity measures and separation techniques [13]. Further, finding an optimal clustering algorithm for a specific bioinformatics problem is challenging and can be formulated as a multi-objective optimization problem [14]. Accordingly, over the decades, clustering analysis approaches such as hierarchical clustering (HC) [15], centroid-based clustering (CC), distribution-based clustering (DC) [16], density-based clustering (DC1) and self organizing maps (SOMs) [17] have been proposed in the literature [1, 7, 13, 18]. Other approaches include probabilistic clustering, grid-based clustering, spectral clustering and non-negative matrix factorization [3]. HC algorithms (e.g. agglomerative [19]) involve creating clusters having a predetermined ordering (top-down or bottom-up), where lower-level clusters are merged into even larger clusters at higher levels, giving a hierarchy of clusters. In agglomerative clustering (AC), initially, each data point is considered an individual cluster. Similar clusters are then merged with other clusters until one or K clusters are formed in each iteration. Advantages of HC algorithms lies in their simplicity and visual appeal, and depending on the desired granularity, one can choose to ‘cut’ the hierarchy at the desired level to obtain a suitable clustering. However, clustering quality (CQ) is sensitive to noise [1], which complicates the interpretation of the hierarchy. Besides, data points are clustered with local decisions based on deterministic attributes, with no chance to reexamine the clustering [1, 7].

In contrast, CC algorithms (e.g. K-means [16], partitioning around medoids (K-medoids) [20]) offer several advantages over SOM and HC [21], e.g. often they obtain superior performance in terms of point density accuracy, topology preservation and computational requirements. However, CC algorithms are incapable of finding non-convex clusters [18]. DC algorithms [e.g. Gaussian mixture model (GMM) [22]] are based on distribution models, where clusters are defined as data points belonging to the discovered distribution(s). In general, DC approaches produce complex models for clusters, hence correlation and dependencies between biological attributes can be captured. The downside is that concisely defined model cannot be developed if the Gaussian distributions are based on a strong assumption on the data. Further, DC algorithms inherently suffer from overfitting issues, unless constraints are put on the model complexity. In DC1 algorithms (e.g. DBSCAN and it’s generalization OPTICS), clusters are defined as areas of higher density compared to the remainder of the dataset; objects in sparser regions are usually considered noise or border points. The critical drawback of DBSCAN and OPTICS, however, is that both expect density drop to detect cluster borders, where GMM based on expectation minimization (EM) can precisely overcome this limitation. Table 1 summarizes different ML-based clustering algorithms showing their strengths and potential limitations.

**Table 1:** Machine learning-based clustering analysis approaches, their potential limitations and bioinformatics use cases

**Algorithm**	**Parameters**	**Scalability**	**Geometry**	**Bioinformatics use cases**	**Limitations**
K-means	Number of clusters	Scalable to a large number of samples	Distances between points	Suitable for a dataset having an even number of cluster size, the flat geometry and not too many clusters; particularly applied for i) clustering microarray and GE data, data visualization and biomedical text clustering	i) Clustering results may differ for a different initial value of K (being the only hyperparameter in K-means), based on the assumption that each cluster is equal-sized and clusters to have hyper-sphere shapes. Hence, density-based clusters cannot be represented, sometimes unstable and profoundly affected if samples are scattered [13], sensitive to noise and outliers thus can get trapped in a local optimum.
DBSCAN	Neighborhood size	Scalable to a large number of samples	Distances between nearest points	Good for uneven cluster size with non-flat geometry, where there are not too many clusters; particularly applied for (i) protein motif [32] and protein sequence clustering [33], (ii) magnetic resonance imaging (MRI) brain tumor segmentation [34] and (iii) gene clustering [35]	(i) Results low-quality (LQ) clustering as it struggles at separating nearby clusters and (ii) quadratic computational complexity, i.e. $O(n^4)$
OPTICS (an extension of DBSCAN)	Minimum cluster membership	Scalable to a large number of samples across clusters.	Distances between points	Suitable for an uneven cluster size with non-flat geometry and variable cluster density; particularly applied for (i) protein motif clustering [32], (ii) clustering of protein sequences [33], (iii) MRI brain tumor segmentation [34] and (iv) gene clustering [35]	(i) Results LQ clustering as it struggles at separating nearby clusters and (ii) quadratic computational complexity, i.e. $O(n^4)$
GMM	Multiple parameters, e.g. number of mixture components, covariance type, convergence threshold and number of EM iterations to perform	Scales poorly for big data having a large number of samples	Mahalanobis distances to centers	Suitable for datasets having a flat geometry and efficient for the density estimation; applied more frequently for (i) GE transformation [36] and (ii) gene clustering	When the number of data points per mixture is insufficient, estimating the covariance matrices becomes difficult. Besides, GMM is known to diverge and find solutions with infinite likelihood unless one regularizes the co-variances artificially. Even though GMM is the fastest algorithm for learning mixture models, it may encounter difficulties at optimizing and model selection on data with complex structure (because GMM requires all the components in which the held-out data or information theoretical criteria is required to decide how many components to use in the absence of external cues).
AC	Number of clusters or distance threshold, linkage type and distance	Scalable to a large number of samples across clusters	Any pairwise distance	Performs better when the input data has many clusters with possible connectivity constraints and non-Euclidean distances; applied more frequently for gene clustering	Although AC can scale up to a large number of samples when jointly used with a connectivity matrix, it is computationally expensive when no connectivity constraints are added between samples. Eventually, AC encounters difficulty at optimizing and model selection for datasets having complex structures.
Partial mixture model (PMM)	Number of clusters or distance threshold, linkage type and distance	Scalable to a large number of samples across clusters	Any pairwise distance	Gene clustering from microarray and GE data where (i) a large number of scattered genes are involved, (ii) there are complex gene inter-correlations and (iii) there is a lack of prior knowledge of the exact number of clusters [13, 37]	PMM requires heavy computation due to repeated sub-sampling. Besides, it may result to slightly different results across two different runs.

Albeit these, algorithms work reasonably well for medium-scale and low-dimensional data, accuracy and efficiency for the high-dimensional datasets having a massive number of samples degrade drastically, mainly due to the *curse of dimensionality*. Besides, ML-based methods generally suffer from high computational complexity on large-scale data [2]. To mitigate the computational complexity, representation learning (RL) is extensively used alongside clustering, to map the input data into a feature space where separation is more straightforward concerning the problem’s context [23]. On the other hand, while dimensionality reduction (DR) using linear transformation methods [e.g. principal component analysis (PCA) [24]], non-linear transformation (e.g. kernel methods [25]) and spectral methods [26] are employed. As observed, clustering with PCA instead of using original variables does not necessarily improve but often degrades the CQ [27]. The reason is that PCA is fundamentally limited to linear embedding, and essential features are often lost [3]. Hence, for better clustering results, high-dimensional datasets require non-linear and spectral DR, without losing important features.

On the other hand, only the fundamentals of deep learning (DL) are currently actively used [28] in bioinformatics research, especially for supervised learning tasks, where RL based on DR and clustering are treated separately and sequentially applied to the data. However, assume, for example, from a large collection of unlabeled images, how to divide them into K-groups in terms of inherent latent semantics? Using an ML-based approach one would (i) first, extract feature vectors according to domain-specific knowledge and (ii) then employ clustering algorithm on the extracted features to group them [29]. In contrast, DL-based approaches can be more effective at RL and feature extraction from the images, which can be used to refine clustering with an auxiliary target distribution derived from the current soft cluster assignment and iteratively improve the clustering [2, 30]. In particular, with a deep neural network (DNN) architecture [e.g. autoencoders (AEs)], more complex and higher-level features can be embedded from the input data, and contextual information can be captured [31]. Eventually, learning non-linear mappings allows transforming input data into more clustering-friendly representations in which the data is mapped into a lower-dimensional feature space [2, 23]. Hence, the cluster assignments can be done with a base clustering algorithm, while iteratively optimizing the clustering objective [23].

Previous reviews [2, 23] on DL-based clustering analysis approaches focused mainly on general-purpose clustering from the perspective of network architectures. In this paper, we provide a comprehensive review of the state-of-the-art DL-based approaches, summarize advantages and point out their potential limitations. Further, we evaluate clustering performance on three bioinformatics use cases—bioimaging, cancer genomics and biomedical text clustering—and provide comparative analyses. To the best of our knowledge, we are the first to review unsupervised DL-based clustering analysis techniques for bioinformatics research. The rest of the paper is structured as follows: Section DL for clustering discusses neural network architectures for clustering analysis, working principles and formulating metrics for evaluating the quality of clustering; Section Evaluation and comparative analysis provides evaluation and comparative analysis of the approaches studied; and Section Conclusions and outlook summarizes the study reported and discusses future work before concluding the paper.

## 2 DL for clustering

As summarized in Table 2, several DL-based clustering analysis approaches have been proposed in the literature. These approaches can be categorized into two leading families: (i) pipeline methods for learning representation using DNN architectures [e.g. multilayer perceptrons (MLPs), convolutional neural networks (CNNs), deep belief networks (DBNs), generative adversarial networks (GANs) [38], variational autoencoders (VAEs), denoising autoencoders (DAEs) and adversarial autoencoders (AAEs) [39]] and clustering using an ML-based clustering algorithm (refer to Table 1 for details) and (ii) single-model methods for end-to-end clustering [2].

**Table 2:** Comparison of DL-based approaches based on the building blocks for clustering analysis (loosely based on [2, 23, 29, 40, 41, 47–50])

**Method**	**Architecture**	**Deep features**	**Non-clustering loss**	**Clustering loss**	**Network update**	**Clustering algorithm**	**Main contributions, robustness and suitability**
DEC [40]	MLP	Encoder output	RL1	CAHL	Pre-training and fine-tuning	Centroid updates and assignments	First well-known DL-based clustering algorithm, easy to implement, particularly suitable for LQ or medium-quality (MQ) imaging (e.g. MNIST, CIFAR-10) with limited data
DCN [41]	MLP	Encoder output	RL1	K-means loss	Joint training and cluster updates	K-means	Clustering using the K-means algorithm and feature learning are done simultaneously. Robustness lies in its simplicity but effectiveness for LQ bioimaging.
IMSAT [50]	MLP	MLP output	IMSAL	-	Joint training	Soft cluster assignments	Introduced self-augmenting in DL-based clustering; particularly, suitable for numeric data & LQ imaging
NMMC [44]	DBN	Last DBN layer	Pre-training loss	MMMC	Pre-training and fine-tuning deepest layer	-	It was introduced to perform non-parametric clustering under a maximum margin, which is a discriminative clustering model. Accordingly, NMMC can learn deep features for clustering numeric data comfortably and reduce model complexity.
UMMC [51]	DBN	Last DBN layer	Pre-training loss	LPL + CAHL	Pre-training and joint training	K-means	The CNN-based joint clustering and optimizing RL1 with feature drift compensation makes it suitable for high-quality (HQ) imaging.
DCC [49]	AE	Encoder	RL1	CAHL	Pre-training and fine-tuning	-	DCC is rooted in robust continuous clustering [52] with a clear continuous objective, where no prior knowledge of cluster number is required. Hence, fine-tuning an extra hyperparameter is not required to find the optimal number of clusters.
VaDE [53]	VAE	Encoder output	ALWMMFC	-	-	Network estimates centroids	Introduced VAE in DL-based clustering, which enforces LFs to follow a predefined Gaussian distribution. Accordingly, the flexibility and scalability of the base networks is increased and the CQ is enhanced. Particularly, VaDE is suitable for clustering numeric data given that computational complexity is not a major concern.
JULE [41]	CNN	CNN output	-	Agglomerative loss	-	AC	Introduced joint unsupervised learning for deep representation of images, making it suitable for bioimaging, albeit computational complexity and memory usage for large-scale images are high
CCNN [45]	CNN	CNN layer	-	CC loss	-	K-means	Requires optimizing only the clustering classification loss, which makes it computationally efficient and, hence, suitable for large-scale imaging and multimedia analytics (e.g. speaker identification).
NNCPC [42]	CNN	CNN output	Cross-entropy loss	-	-	K-means	This work introduced a strategy to employ contrastive criteria that pushes data-forming clusters directly from the input data, in addition to learning a feature embedding suitable for clustering LQ images, numeric and text data. Besides, it is resilient to noise and largely insensitive to the number of clusters.
DEPICT [46]	CNN	Encoder output	RL1	Balanced assignment	Pre-training and joint training	Centroid updates and assignments	Can extract quality and deep features from LQ/MQ/HQ images. It is computationally efficient and robust, making it suitable for large-scale imaging.
DBC [47]	CNN	Encoder output	RL1	CAHL	Pre-training and fine-tuning	K-means	It improves the limitations of DEC by introducing CNN making it suitable for LQ/MQ/HQ imaging.
CEN [30]	CAE	Encoder output	RL1	CAHL	Pre-training and joint training	K-means	Can extract quality and deep features from HQ images as well numeric data. It is computationally efficient and robust making it suitable for large-scale imaging, numeric and genomics data.
DCEN [29]	CAE	Encoder output	RL1	CAHL	Pre-training and joint training	K-means	Can preserve the intrinsic structure of data-generating distribution and help clustering loss to manipulate the embedded feature space appropriately. Suitable for LQ/MQ imaging data.
DAC [53]	GAN	Penultimate discriminator layer	RL1	GMM likelihood and adversarial objective	K-means++	Network predicts soft assignments to clusters	Inspired by VAE, adversarial AE is introduced in which an adversarial training procedure is followed to match the aggregated posterior of the LFs with the prior distribution, making it suitable for HQ imaging.

However, pipeline methods are extensively discussed in the literature. Examples include deep embedding clustering (DEC) [40], deep clustering network (DCN) [41], clustering using pairwise constraints clustering CNN (NNCPC) [42], deep embedding network (DEN) [43], joint unsupervised learning of deep representation for images (JULE) [41], DL with non-parametric clustering (NMMC) [44], clustering using CNN (CCNN) [45] and deep clustering with convolutional autoencoder (CAE) embedding (DEPICT) [46].

More recent approaches include convolutional embedded networks (CENs) [30], deep convolutional embedded clustering (DCEN) [29], discriminatively boosted clustering (DBC) [47], CNN-based joint clustering and RL with feature drift compensation (UMMC) [51], deep continuous clustering (DCC) [49], learning latent representations for clustering (IMSAT) [50] and deep adaptive clustering (DAC) [48]. We listed reviewed approaches including links to the original papers and their implementations that can be found at https://github.com/rezacsedu/Deep-learning-for-clustering-in-bioinformatics.

### 2.1 Working principles of DL-based clustering methods

Let us consider the problem of clustering of $n$ samples, $X$ = $\{\mathbf{x}^{(1)},\mathbf{x}^{(2)},..., \mathbf{x}^{(n)}\}$ into $K$-categories, each represented by a centroid $\mu _{j}, j=1, \dots , K$ where $X \in \mathbb{R}^{D}$. In pipeline methods, more or less a similar working principle is followed in which a DL-based clustering algorithm is usually trained in two phases:

$\bullet $  **Phase 1:** parameter initialization and RL with a DNN architecture and training using *non-clustering* loss (e.g. standard RL1). Then clustering-friendly representations of the data called latent features (LFs) are extracted from one or more layers (depending on the type of network architecture).
$\bullet $  **Phase 2:** parameter optimization by iterating between computing an auxiliary target distribution and minimizing *clustering* loss [e.g. Kullback–Leibler divergence (KLD) [54] and cluster assignment hardening loss (CAHL)] in which cluster assignments are formulated, followed by the centroid updated with the backpropagation in which an ML-based clustering algorithm is applied to optimize the clustering objective iteratively. In particular, AC [41] and K-means [42, 48, 51] algorithms are broadly used in the literature [2].

Followed by this principle, instead of clustering the samples directly in the original input space $X$, it is transformed with a nonlinear mapping $f_{\theta }: X \rightarrow Z$ where $\theta $ are learnable parameters and $Z \in \mathbb{R}^{K}$ is the learned or embedded feature space, where $K \ll D$. To parametrize $f_{\theta }$, a DNN architecture such as AEs is used due to their function approximation properties and feature learning capabilities [40] (refer to Section 2.2). However, for a better clustering result, the network is often trained and updated to optimize both clustering and non-clustering losses jointly in phase 2. Concisely, the following three steps are broadly involved in existing approaches:

$\bullet $ RL by embedding a higher-dimensional input space into a lower-dimensional feature space to generate cluster-friendly features using a neural network architecture;
$\bullet $ Combining clustering and non-clustering losses;
$\bullet $ DNN and clustering algorithm’s parameters updates to optimize the combined loss.

### 2.2 RL with DNNs

Good clustering accuracy can only be attributed to the fact that multiple network layers are stacked together in which weights are reused in subsequent layers for the RL [23]. Since most of the state-of-the-art approaches used AEs, we avoid the details of MLP, CNN and DBN-based RL. A regular AE consists of multi-layer dense networks called encoder and decoder, which is architecturally an MLP. First, the encoder learns the representation of input $x$ in a compressed format in which the data is mapped and transformed into an embedding $z$. Then the decoder tries to reconstruct $x$ from $z$ by reducing the reconstruction loss (RL1) between $x$ and its corresponding reconstruction $x^{\prime } \in \mathbb{R}^{D}$ such that useful information is not lost in the encoding phase [54]. Usually, RL1 is the distance measure ($d_{AE}$) between input $x_i$ and network’s output $f(x_i)$:

(1) \begin{equation*} L_{AE}=\text{{$d_{AE}$}}(x_i, f(x_i) = \sum_{i} ||x_{i}-f(x_i)||^{2}. \end{equation*}

#### 2.2.1 Data preprocessing

In bioinformatics research, a large variety of datasets are curated from multiplatform (e.g. TCGA provides DNA methylation data curated from HumanMethylation450 and HumanMethylation27 platforms) and heterogeneous sources (e.g. similar types of data curated from Broad Institute, the Massachusetts Institute of Technology and Harvard) need to be dealt with. Further, since clustering algorithms are used to discover hidden patterns from the data, CQ depends on the distributions of the data points and the underlying representation [40]. Therefore, depending on problems and data types, different types of preprocessing may require depending upon DNN architectures. For example, genomics data like GE can be represented using log-transformed expression values [e.g. $\log _{2}(FPKM+1)$, where FPKM (a normalized estimation of GE-based on RNA-Seq data) is the number of fragments per KB per million mapped reads [56]] to model proportional chances rather than additive changes, which is biologically more relevant. This way, genes with low information burden (e.g. $mean<0.4$ or SD $< 0.75$) across all the samples can be removed.

In bioimaging, different types of preprocessing required depending on modality types in order to mitigate bottlenecks like noise and artifact, e.g. rescaling, horizontal flipping and enhancement using histogram equalization and slight Perona–Malik filtering for radiographs, while MRIs require contrast enhancement, intensity regulation and noise elimination [57]. Moreover, approaches such as DEC expects normalized input such that $\frac{1}{d}||z_i||^{2}_{2}$ is approximately 1, where $d$ is the dimension of the data space $\lbrace{z_{i}\in Z}\rbrace $ [58].

#### 2.2.2 Extracting cluster-friendly deep features

In the context of clustering, after the training, the decoder part of an AE is no longer used but only the encoder is left, which acts as the feature extractor. LF then can be extracted from one or more layers (depending on the type of network architecture). For example, if extracted from a single layer, features come typically from the last layer of the network. However, if extracted from a multilayer or deep network (from any hidden layer or the deepest layer), it is found that LF can lead to better feature representations that can enhance the separation of data points during the similarity computation [59]. Table 3 provides a short overview of different feature extraction methods, showing their advantages and potential limitations.

**Table 3:** Comparison of the feature extraction process in DL-based clustering algorithms

**Feature extractor**	**Advantages**	**Disadvantages**
AE	One of the simplest and MLP-based autoencoding techniques. It learns hidden features to encode and decode the data without considering the probability distribution of the input samples. Hence, it is easy to implement and extract features from the encoder component.	AEs have a huge number of hyperparameters, which is why it is tricky to optimize and balance between clustering and non-clustering losses. Since it learns the hidden representation discriminatively to encode and decode the data blindly using a shallow network architecture. A fundamental problem with an AE is with the LF it embeds their inputs to and where their encoded vectors lie, may not be continuous and may allow easy interpolation. Consequently, CQ would be poor in the case of bioimaging and biological sequence data. Although the computational complexity depends on the clustering loss, it requires many iterations to optimize a large number of hyperparameters.
DBN	A simple generative model based on RBM, which has very rich mathematical and conceptual justification in its structure as well as its training algorithms. Works moderately well even in a limited labeled dataset because it can be pre-trained in an unsupervised way, and the pre-training weights can be reused for a supervised learning task.	DBN-based RL has a risk of obtaining a corrupted LF space if the RBM pretraining loss goes out of bounds. Further, to avoid overfitting, it typically requires many samples to train well.
CNN	Has a straightforward graceful objective, hence can be extended to large-scale clustering tasks. Deep and quality features can easily be extracted for numerous bioinformatics use cases, e.g. bioimaging, text (i.e. sequence) clustering and genomics. It has a fewer number of hyperparameters than a regular AE or VAE, which makes it easier to optimize the overall network.	Since there is a risk of obtaining a corrupted LF space, a well-defined clustering loss is required to balance between clustering and non-clustering losses, which is tricky. To avoid overfitting, CNN typically requires many samples to get trained well.
CAE	Has straightforward graceful objective, hence can be extended to large-scale clustering tasks. Deep and quality features can be easily extracted for bioimaging and text clustering. Further, since in CAEs, weights are shared among all locations in the input, preserving locality and reducing the number of parameters than regular AEs, VAEs and CNNs [61].	Since there is a risk of obtaining a corrupted LF space, a well-defined clustering loss is required to balance between clustering and non-clustering losses, which is tricky. Similar to CNN, CAE also requires many samples to be trained well to avoid overfitting.
VAE	Capable to generate artificial samples, which makes it suitable for bioinformatics use cases with limited labeled or unlabeled samples. Particularly suitable for numeric and genomic data. Besides, it has a decent theoretical guarantee and mathematical interpretation.	The computational complexity is very high, hence requires many iterations to optimize numerous hyperparameters. Exhibits poor clustering in the case of HQ bioimaging.
AAE	Capable to generate artificial samples, which makes it suitable for bioinformatics use cases with limited labeled or unlabeled samples. Particularly suitable for numeric and genomic data. Besides, the flexible nature of GAN and its variants can be used to disentangle both discrete and continuous latent factors. Hence, it can scale to complex datasets.	Since AAE’s optimizing objective comprises several losses (i.e. RL1, GMM likelihood, and adversarial objective), computation complexity is very high and hard to converge.

#### 2.2.3 Enhancing robustness of RL

In some early approaches (e.g. NMMC and UMMC) DBN is employed as the feature extractor in which Restricted Boltzmann Machines (RBM) [60] formed the basic building block. However, despite numerous successes, DBN has gradually been replaced with AE. On the other hand, although used for clustering numeric data and LQ images, AE is mostly not suitable for 2D/3D finite and discrete signals or digital images [2], primarily because of their weak RL capability. Subsequently, several ways by employing different DNN architectures have been proposed to improve the quality of the RL [2, 23]:

****CNN and CAE:**** since a vanilla AE is not suitable for handling data with spatial invariance (e.g. HQ images), they are incapable of preserving spatial relationships between pixels in an object. However, CNN can be a better feature extractor as it can preserve local structure in which output from the deepest convolutional (conv) layer can be extracted as LF (e.g. JULE). On the other hand, instead of manually engineered conv filters, conv and pooling layers can be added to construct a CAE, where each layer consists of an encoder (that performs convolution and pooling operations), and a decoder (to perform unpooling and deconvolution operations), and a conv layer of the encoder calculates the $j^{th}$ feature map as follows [62]:

(2) \begin{equation*} h^{j}=\sigma\left(x_{i} * W_{ij}^{j}+b^{j}\right), \end{equation*}

where $x_i$ is the input sample, $W_{ij}^{j}$ is the $j^{th}$ filter from input channel $i$ and filter $j$, $b^j$ is the bias for the $j^{th}$ filter, i.e. single bias per latent map (one bias per GV would introduce many degrees of freedom), $\sigma $ is an activation function [i.e. rectified linear unit (ReLu)] and * denotes the conv operation. To obtain translation-invariant representations, max-pooling is performed by downsampling conv layer’s output and taking the maximum value in each $m \times n$ non-overlapping sub-region [62]. In the decoding phase, unpooling and deconvolution operations performed to preserve the positional-invariance information during the pooling operations. Then the deconvolution operation is performed to reconstruct $x_i$ as follows [62]:

(3) \begin{equation*} x_i = \sigma\left(o^{j} * W_{oj}^{j}+c^{j}\right), \end{equation*}

where $o^j$ is $j^{th}$ feature map and $W_{oj}^{j}$ is $j^{th}$ filter of unpooling layer $o$; $j$ and $c^j$ are filter and bias of $j^{th}$ output layer, respectively. This way, compared to CNN (e.g. DBC, CEN, DCEN and DEPICT), CAE learns optimal filters and minimize the RL1, which results in more abstract features from the encoder (e.g. pixel-level features from images) that help to stabilize training and network converges faster, avoid corruption in feature space and improve the CQ [29]. Besides,ref40, VAE or LSTM-AE can be constructed in different scenarios (e.g. imaging, sequence) for the RL.

****VAE:**** generative variants of AE are also used in literature (e.g. VaDE [53]) in combination with a mixture of Gaussian. VAE enforces the latent code of AE to follow a predefined distribution, which combines variational Bayesian methods and increases the flexibility and scalability of the base network. Architecturally, VAE is different compared to AE or CAE and deeply rooted in the methods of variational Bayesian and graphical models, where the input is into distribution, as shown in Figure 3. This distribution, say $p_{\theta }$, is parameterized by $\theta $, where $p_{\theta }(\mathbf{z})$ is the prior, $p_{\theta }(\mathbf{x} | \mathbf{z})$ is the likelihood, and $p_{\theta }(\mathbf{z} | \mathbf{x})$ is the posterior given that the real parameter $\theta ^*$ is known for the distribution.

**Fig. 1:**
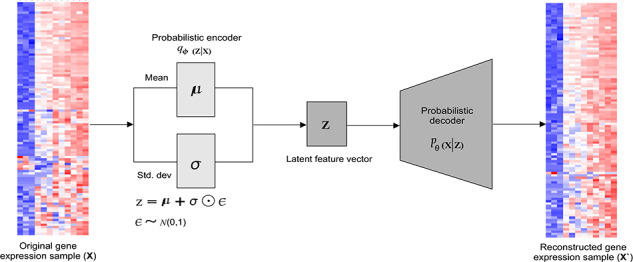
An example of clustering microscopy image with CEN in which a CAE is used for the RL. LFs are then extracted and fed into a base clustering algorithm for the soft clustering assignment. Finally, the RL of CAE (blurred image signifies the existence of RL1) and CAHL of base clustering algorithm are optimized jointly through backpropagation.

To generate a sample similar to a real data point $\mathbf{x}^{(i)}$: (i) first, $\mathbf{z}^{(i)}$ can be sampled from a prior distribution $p_{\theta ^{*}}(\mathbf{z})$; (ii) then, $\mathbf{x}^{(i)}$ can be generated from the conditional distribution $p_{\theta ^{*}}\left (\mathbf{x} | \mathbf{z}=\mathbf{z}^{(i)}\right )$, $\theta ^{*}$ is the optimal parameter that maximizes the probability of generating real data samples [53]:

(4) \begin{equation*} \theta^{*}=\arg \max _{\theta} \sum_{i=1}^{n} \log p_{\theta}\big(\mathbf{x}^{(i)}\big) \end{equation*}

 

(5) \begin{equation*} p_{\theta}\big(\mathbf{x}^{(i)}\big)=\int p_{\theta}\big(\mathbf{x}^{(i)} | \mathbf{z}\big) p_{\theta}(\mathbf{z}) \textrm{d} \mathbf{z} \end{equation*}

The data generation process involving the encoding vector can be expressed in (5) [53]. Eventually, VAE consists of a probabilistic encoder as an approximation function $q_{\theta }(\mathbf{z} | \mathbf{x})$ (which is similar to $g_{\phi }(\mathbf{z} | \mathbf{x})$) and a generative probabilistic decoder as the conditional probability $p_{\theta }(\mathbf{x}|\mathbf{z})$ (which is similar to the decoder $f_{\theta }(\mathbf{x}|\mathbf{z})$). In variational inference, objective is to maximize the variational evidence lower bound (ELBO) by maximizing the lower bound (‘lower bound’ comes from the fact that KL divergence is always non-negative, hence $-L_{vae}$ is the lower bound of $\log p_{\theta }(\mathbf{x})$) as follows [53]:

(6) \begin{equation*} -L_{vae}=\log p_{\theta}(\mathbf{x})-L_{\mathrm{ref55}}\left(q_{\phi}(\mathbf{z}|\mathbf{x}) \| p_{\theta}(\mathbf{z}|\mathbf{x})\right) \leq \log p_{\theta}(\mathbf{x}). \end{equation*}

VAE and its variants (e.g. LSTM-based VAE [63]) are used in across use cases such as anomaly detection. In which anomalous or outliers can be identified based on the reconstruction probability (RP) [64], which is a probabilistic measure that takes into account the variability of the distribution of variables. Since RP has a theoretical background, it is a more principled and objective anomaly score than the RL1.

****LSTM-AE:**** VAE or CAE are not the best options for handling sequence or time-series data, e.g. length of the input sequence in case of text clustering may vary while the network requires fixed-length inputs. Further, the temporal ordering of the observations makes the feature extraction difficult. Hence, regular AEs will fail to generate a sample sequence for a given input distribution in generative mode, whereas LSTM-AE can handle variable lengths as the encoder learns fixed-length vector representation of the input [65, 66].

**Fig. 2:**
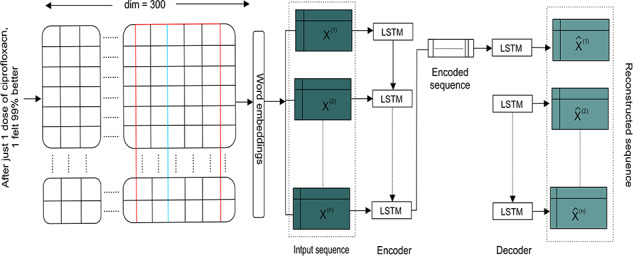
Schematic representation of the LSTM-AE, used for biomedical text clustering, where individual drug review texts are embedded using word2vec before feeding as a sequence.

Given $X$ = $\{\mathbf{x}^{(1)},\mathbf{x}^{(2)},..., \mathbf{L}^{(1)}\}$ a input sequence, $\mathbf{h}_E^{(i)} \in{R}^c$ is encoder’s hidden state at time $t_i$ for each $i \in \{1,2,...,L\}$, and $c$ is the number of LSTM units [67]. The encoder and decoder are jointly trained to reconstruct the original vector in reverse order by minimizing the following objective [68]:

(7) \begin{equation*} \sum_{X \in S_n}\sum_{i=1}^{L}\|\mathbf{x}^{(i)}-\mathbf{x}^{\prime (i)}\|^2, \end{equation*}

where $S_n$ is a set of training sequences. The final state $\mathbf{h}_E^{(L)}$ of the encoder is used as the initial state for the decoder. The the decoder uses $\mathbf{x}^{(i)}$ as input to obtain state $\mathbf{h}_D^{(i-1)}$ and predict $\mathbf{x^{\prime}}^{(i-1)}$ corresponding to target $\mathbf{x}^{(i-1)}$ [67] as shown in Figure 2.

**Fig. 3:**
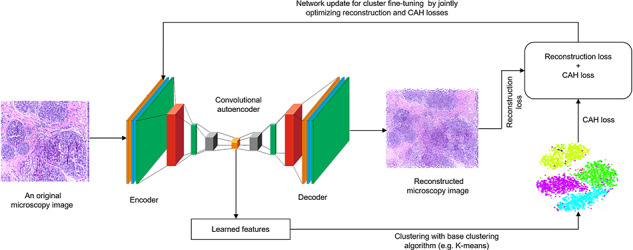
Schematic representation of a VAE used for clustering GE data, where an individual GE sample is fed into the model for learning representation.

****AAE:**** in more recent approaches, adversarial AE is employed in which the adversarial training procedure is followed to match the aggregated posterior of the latent representation with the prior distribution. Thus, AAE can be used to generate artificial samples for bioinformatics use cases with a limited number of labeled or unlabeled (i.e. numeric or genomic) data, where the flexible nature of GAN can be utilized to extract discrete and continuous LF from large-scale data [2].

In particular, information maximizing generative adversarial network (a.k.a. InfoGAN) [69] is used for optimizing the mutual information between a fixed small subset of the GAN’s noise variables and the observation [70], assuming (i) computation complexity is not a prime concern and (ii) appropriate hyperparameters can be found.

****DAE:**** since a good representation is one that can be obtained robustly from a corrupted input, and that will be useful for recovering the corresponding clean input [71], to improve the robustness of the RL, features noise can be introduced to the input [2]. As a result, denoising helps the AE to learn the LFs present in the data. In particular, DAEs take a partially corrupted input, learns a vector field for mapping the input data into a lower-dimensional manifold ($f_{\theta }$) in order to recover the original undistorted input by (i) ignoring the added noise and (ii) by minimizing the RL1 between reconstructed and the original input:

(8) \begin{equation*} L_{DAE} = |\tilde{x}-g(f(x))|. \end{equation*}

For bioimaging, however, convolutional denoising autoencoder (CDAE) can be used to denoise corrupted images, which then ensures a good representation is one that can be derived robustly from a corrupted input to be used for recovering the corresponding clean input.

****Stacked AE:**** The input can be denoised and passed through by stacking autoencoders (SAE), e.g. where the input corruption is used only for the initial denoising. Once the mapping function $f_{\theta }$ is learned, the uncorrupted input from the previous layers are reused in the subsequent layers, e.g. DEC initializes the network with SDAE, where each layer is a DAE trained to reconstruct previous layer’s output after random corruption (i.e. DAE). Intuitively, such an SDAE can be considered a two-layer network and formulated as follows [40]:

(9) \begin{eqnarray*} \tilde{x}\sim dropout(x) \end{eqnarray*}

 

(10) \begin{eqnarray*} h = g_{1}(W_{1}\tilde{x}+b_{1}) \end{eqnarray*}

 

(11) \begin{eqnarray*} \tilde{h}\sim dropout(h) \end{eqnarray*}

 

(12) \begin{eqnarray*} y = g_{2}(W_{2} \tilde{h}+b_{2}), \end{eqnarray*}

where $dropout(.)$ is the dropout operation [72], $g_1$ and $g_2$ are activation functions for encoding and decoding layer, respectively, and $\theta $=$\lbrace{W_1, b_1, W_2, b_2}\rbrace $ are model hyperparameters [40]. Then greedy layer-wise training (GLW) is performed by minimizing the least-squares loss $||x-y||^{2}_{2}$, i.e. after training one layer, output $h$ is used as the input to the next layer and so on. In such a scenario, ReLU activation function is used in all encoder and decoder pairs, except for $g_2$ (first pair) and $g_1$ (last pair).

Once the GLW training is finished, all the encoder and decoder layers are concatenated in reverse layer-wise training order, by forming a deep AE and fine-tuned to minimize the RL1. During the GLW pretraining, each layer is pretrained for a relatively higher number of iterations with a dropout. The result is a multilayer deep AE with a bottleneck-coding layer in the middle. Based on a similar principle, other types of AE can be stacked to form such a deep AE architecture.

### 2.3 Network updates and training

Training DL-based clustering algorithms may vary depending on the DNN architecture, different loss functions and training methods. However, since covering each of them in complete detail would be cumbersome in this comparative analysis, we discuss the detail of network updates and training for the pipeline methods (e.g. DEC) only that includes most of the possible steps explained in other DL-based approaches. In DL-based clustering, following two types of losses are optimized (interested reader can refer to the literature [2, 23] for the details of these loss functions):

$\bullet $  **Non-clustering loss**: this types of losses (e.g.[54], CAHL, K-means loss, balanced assignments loss, locality-preserving loss, group sparsity loss and AC loss) are independent of the clustering algorithm and usually enforces a desired constraint on the learned model, which guarantees that the learned representation preserves important information (e.g. spatial relationships between features) so the original input can be reconstructed in the decoding phase [2].
$\bullet $  **Clustering loss**: this type of loss (e.g. RL1 and self-augmentation loss) is specific to the clustering method and the clustering-friendliness of the learned representations [23].

In phase 1, RL1 of the AE is minimized. Once RL1 of the decoder module is optimized, the decoder module is no longer used, but only the LF are extracted from encoder with a bottleneck-coding layer in the middle. Then from an initial estimate of the non-linear mapping $f_\theta $ and initial centroids $\lbrace{\mu _{j}\in Z}\rbrace ^{K}_{j=1}$ (as trainable weights $Z$), clustering can be improved by alternating between two steps as in the literature [40]:

$\bullet $  **Step 1:** soft assignment of $Z$ to the cluster centroids;
$\bullet $  **Step 2:** updating the mapping $f_\theta $ and refining cluster centroids by learning from initial assignments using an auxiliary target distribution.

These steps are repeated until a convergence criterion is met. Initializing clustering on LF generates the second type of loss called CAHL, which is specific to the clustering method and clustering-friendliness of the learned representations [23]. Similar to literature [40], we considered normalized similarities between data points and centroids as soft cluster assignments in which Student’s *t*-distribution [73] is used as a kernel to measure the similarity between embedded point $z_j$ and centroid $\mu _j$, where $z_i$= $f_\theta $  $(x_i) \in Z$ corresponds to $x_i$  $\in X$ after embedding, $\alpha $ is the degree of freedom, and $q_{ij}$ is the probability of assigning sample $i$ to cluster $j$ [40].

(13) \begin{equation*} q_{ij}=\frac{(1+||z_{i}-\mu_{j}||^{2}/\alpha)^{-\frac{\alpha+1}{2}}}{\sum_{j^{\prime}}(1+||z_{i}-\mu_{j^{\prime}}||^{2}/\alpha)^{-\frac{\alpha+1}{2}}} \end{equation*}

However, cross-validation of $\alpha $ in the unsupervised setting is not a viable option. Moreover, learning $\alpha $ is superfluous, similar to literature [40], we set $\alpha $ to 1. In step 2, the similarity between the distributions is evaluated using KLD w.r.t. by decreasing the distance between soft assignments ($q_{ij}$) and the auxiliary distribution ($p_{ij}$) as follows [40]:

(14) \begin{equation*} L_{ref55}=\textrm{KL}(P||Q)=\sum_{i}\sum_{j}p_{ij}\log\frac{p_{ij}}{q_{ij}}, \end{equation*}

where $q_{ij} \in Q$ and $p_{ij} \in P$ are optimized through backpropagation. Minimizing this loss w.r.t. network parameters lead to smaller distances between the data points and their assigned cluster center for a better CQ, where the loss is computed by favoring a situation where points of a cluster are close to the mean of the cluster. Conversely, points that are close to the mean of another cluster will adversely affect the loss. However, since setting $P$ is crucial to increase the CQ, soft assignment $q_{ij}$ is computed by raising auxiliary distribution $p_{ij}$ to the second power and normalizing by frequency per cluster as follows [40]:

(15) \begin{equation*} p_{ij}=\frac{q^{2}_{ij}/f_{j}}{\sum_{j^{\prime}}q^{2}_{ij^{\prime}}/f_{j^{\prime}}}, \end{equation*}

where $f_{j}=\sum _{i}q_{ij}$ are soft cluster frequencies and $P$ forces the assignments to have stricter probabilities between [0–1]. On the other hand, since the constraints enforced by the RL1 can be lost after training the network longer, using only clustering loss may lead to worse clustering results [23]. To tackle this issue, the literature [2, 43, 50] performed joint training by setting $\alpha $ such that the network training is affected by both clustering and non-clustering loss functions simultaneously in which we combine clustering and non-clustering losses with a linear combination of individual loss [23]:

(16) \begin{equation*} L(\delta) = \sigma L_{ref55}(\delta) + (1 - \sigma)L_{AE}(\sigma), \end{equation*}

where $L_{ref55}(\delta )$ is the clustering loss, $L_{AE}(\sigma )$ is the non-clustering loss, and $\sigma \in [[0, 1]]$ is a constant hyperparameter to specify the weighting between both functions. To assign and schedule $\sigma $, following options are employed [40]:

$\bullet $  **Pre-training and fine-tuning phase:**  $\sigma $ is usually set to 0 and network is trained using non-clustering loss only.
$\bullet $  **Afterwards:**  $\sigma $ is set to 1 by removing the non-clustering network branches (e.g. decoder), which ensures the clustering loss to be used to fine-tune the pretrained network.

The combined loss $L(\delta )$ is then optimized using first-order gradient-based optimizers such as Adam, AdaGrad or RMSprop with varying learning rates and different batch size, where gradients of $L$ (w.r.t $Z$) for each data point $z_i$ and cluster centroid $\mu _{j}$ are computed as follows [40]:

(17) \begin{eqnarray*} \frac{\partial L}{\partial z_{i}}&=&\frac{\alpha+1}{\alpha}\sum_{j}\left(1+\frac{||z_{i}-\mu_{j}||^{2}}{\alpha}\right)^{-1} \end{eqnarray*}

 

(18) \begin{eqnarray*} & &\times(p_{ij}-q_{ij})(z_{i}-\mu_{j})\nonumber\\ \frac{\partial L}{\partial \mu_{j}}&=&-\frac{\alpha+1}{\alpha}\sum_{i}\left(1+\frac{||z_{i}-\mu_{j}||^{2}}{\alpha}\right)^{-1} \\ & &\times(p_{ij}-q_{ij})(z_{i}-\mu_{j}) \nonumber. \end{eqnarray*}

Gradients $\partial L$/$\partial z_{i}$ are then passed to the DNN and used in standard backpropagation to compute network’s parameter gradient $\partial L$/$\partial \theta $. This iterative process continues until less than $tol$% of points change cluster assignment between two consecutive iterations for the cluster assignments [40].

### 2.4 Evaluation metrics for clustering

In the literature [23, 30], three empirical approaches are used for determining the optimal number of clusters to be set before training based clustering algorithms. These are Elbow [74], generalizability $G$ and Normalized Mutual Information (NMI) [75]. In Elbow, the cost is calculated using the within-cluster sum of squares (WCSS) as a function of the number of clusters, $K$. Since Elbow performs better in a classical clustering setting [30], for evaluating clustering results with different cluster numbers, NMI is proposed [40], which tells us the reduction in the entropy of class labels we get assuming the cluster labels are known and can be computed as follows:

(19) \begin{equation*} NMI(y, c) = \frac{I(y,c)}{\frac{1}{2}[H(y) + H(c)]}, \end{equation*}

where $y$ signifies the ground-truth labels, $c$ means the cluster assignment from the algorithm, $I$ is the mutual information between $y$ and $c$ and $H(.)$ is then entropy. NMI is a good measure for determining the quality of the clustering. Further, since it is normalized, we can measure and compare the NMI between different clustering having a different number of clusters. For example, if the NMI for the second clustering is higher than the first clustering, we should prefer the second clustering to the first. On the other hand, $G$ is defined as the ratio between training and validation loss [40] as in (20), in which $G$ is small when training loss is lower than the validation loss, an indication of a high degree of overfitting.

(20) \begin{equation*} G = \frac{L_{train}}{L_{validation}} \end{equation*}

Since a good clustering performance is also characterized by high intra-cluster similarity and low inter-cluster similarity for the data points, rand index (RI) is calculated based on the permutation model (PM) as follows:

(21) \begin{equation*} RI = \frac{TP+TN}{TP+FP+FN+TN}, \end{equation*}

where TP, TN, FP and FN signify true positive, true negative, false positive and false-negative rates, respectively. RI, which is used to measure the percentage of decisions that are correct to evaluate the CQ [76], has a value between 0 and 1, where 0 indicates the disagreement between two data clusters on any pair of points and 1 signifies the perfect agreement (i.e. the same cluster). Thus, the higher the RI, the better the CQ is. Since RI was corrected using the PM for clusters in which the number and size of clusters within a clustering are fixed, and all random clusters are generated by shuffling the elements between the fixed clusters, the premises of the PM are frequently violated [77]. For example, in many clustering scenarios, either the number of clusters or the distribution size of the clusters varies drastically [78]. Therefore, RI is normalized to adjusted rand index (ARI) for values between -1 (independent labeling) and 1 (perfect match) [79], even though it is safer to use ARI, especially in case of smaller sample sizes or larger number of clusters.

Further, to evaluate the CQ, unsupervised clustering accuracy (ACC) [40] metric is used, which takes a cluster assignment from an unsupervised algorithm, assigns the ground truths and computes the best match between them. Intuitively, it measures the best matching between cluster assignments from a clustering method and the ground truth. So, given the ground-truth label $y_i$ and the cluster assignment from the algorithm $c_i$, ACC can be computed as follows:

(22) \begin{equation*} ACC = \operatorname*{max}_{m} \frac{\sum\limits_{i=1}^n 1\Bigl\{y_i=m(c_i)\Bigr\}} {n}, \end{equation*}

where $m$ ranges overall possible one-to-one mappings between clusters and labels using Hungarian algorithm [80]. Additionally, if the ground truth class assignments of the samples are given, metrics like homogeneity and completeness can be formulated to desirable objectives for any cluster assignment using conditional entropy analysis [81]. While the former signifies if each cluster contains only members of a single class, the latter signifies if all members of a given class are assigned to the same cluster.

## 3 Evaluation and comparative analysis

To show the effectiveness of DL-based clustering approaches, we focus on clustering genomics data, biomedical text mining and bio-imaging using different methods. Since DEC is based on MLP, for each use case, CAE, VAE and LSTM-AE were trained with five different base clustering algorithms for the soft clustering assignment. For the brevity, however, we demonstrated CAE-, VAE- and LSTM-AE-based RL in detail for bioimaging, GE and text clustering, respectively. Finally, we evaluated the performance of all these approaches both quantitatively and qualitatively and provided a comparative analysis.

### 3.1 Experiment setup

All programs were written in Python, and experiments were carried out on a machine having 32 cores, 256GB of RAM and Debian 9.9 OS, where the software stack consisted of Keras and scikit-learn with the TensorFlow backend. Network training is carried out on an NVIDIA Titan XP GPU with CUDA and cuDNN for faster network training. Interactive Python Notebooks and further technical details can be found at https://github.com/rezacsedu/Deep-learning-for-clustering-in-bioinformatics.

Results based on best hyperparameters produced through random search are reported empirically, where we verified whether the network converges to the optimal number of clusters by setting $K=2$ and increasing it slowly. We also focused on investigating how the network training converged during the cluster assignments and updates by utilizing the Elbow method in which WCSS is calculated along with other metrics such as ARI, NMI, ACC, completeness and homogeneity.

### 3.2 Clustering breast microscopy images

Breast cancer is one of the main causes of death worldwide. However, early diagnosis significantly increases treatment success for which histology images are essential [82]. The diagnosis of biopsy tissue with hematoxylin and eosin-stained images is non-trivial, and specialists often disagree on the final diagnosis. Individually, during the diagnosis procedure, specialists evaluate both overall and local tissue organization via whole-slide and microscopy images [83]. We applied CEN on the Grand Challenge on the BreAst Cancer Histology (BACH) [84] dataset in which images are classified into four classes according to the predominant cancer type in each image: healthy tissue, benign lesion, in-situ carcinoma and invasive carcinoma. The original balanced image dataset is composed of 400 labeled microscopy high-resolution (2040 x 1536 pixels), uncompressed and annotated haematoxylin and eosin stain (H & E) stain images. The annotation was performed by two medical experts, and images with disagreements were discarded.

The schematic representation of the CA E-based clustering is depicted in Figure 1, where the CAE consists of 24 layers. Batch normalization (BN) is used before non-linearities (i.e. conv and dense layers) and ReLU activation function in every layer. During the RL phase, a conv layer of encoder calculates the feature map from a given image and the up-sampling layer scales up the image. Layer-wise structure of the CAE is as follows:

- Input layer: each $2040 \times 1536 \times 3$ microscopy image is reduced to $508 \times 508 \times 3$
- Convolutional layer: of size $127 \times 254 \times 254$
- BN layer: of size $127 \times 254 \times 254$
- Max-pooling layer: of size 2 $\times $ 2
- Convolutional layer: of size $64 \times 127 \times 127$
- BN layer: of size $64 \times 127 \times 127$
- Max-pooling layer: of size 2 $\times $ 2
- Convolutional layer: of size $32 \times 64 \times 64$
- BN layer: of size $32 \times 64 \times 64$
- Max-pooling layer: of size 2 $\times $ 2
- Convolutional layer: of size $32 \times 64 \times 64$
- BN layer: of size $32 \times 64 \times 64$
- Max-pooling layer: of size 2 $\times $ 2
- Upsampling layer: of size 2 $\times $ 2
- Deconvolutional layer: of size $32 \times 64 \times 64$
- BN layer: of size $32 \times 64 \times 64$
- Upsampling layer: of size 2 $\times $ 2
- Deconvolutional layer: of size $64 \times 127 \times 127$
- BN layer: of size $64 \times 127 \times 127$
- Upsampling layer: of size 2 $\times $ 2
- Deconvolutional layer: of size $127 \times 254 \times 254$
- BN layer: of size $127 \times 254 \times 254$
- Upsampling layer: of size 2 $\times $ 2
- Deconvolutional layer: of size $508 \times 508 \times 3$.

Then clustering steps are similar to CAE as discussed before in which we initiate the clustering by setting $K=2$ (where applicable) for the base clustering algorithms and increase it up to 10 to see the performance towards optimal clustering assignments. We observed how the generalizability and WCSS values change for different K values to find the optimal number of clusters. We perform the hyperparameters tuning of each approach separately in which 5-fold cross-validation and random search techniques were employed.

Clustering results for this experiment are reported in Table 4 showing different metrics. In particular, as highlighted in cyan, with the best hyperparameters, AC algorithm performs best based on CAE-based LF, giving an ARI of 0.85, an NMI of 0.83 and an ACC of 0.84 in which each cluster contains only members of a single class in 75% of the cases and in 77% of the cases all members of a given class (either healthy tissue, benign lesion, in-situ carcinoma or invasive carcinoma) are assigned to the same cluster (since ground truth class assignments of the samples are given).

**Fig. 4:**
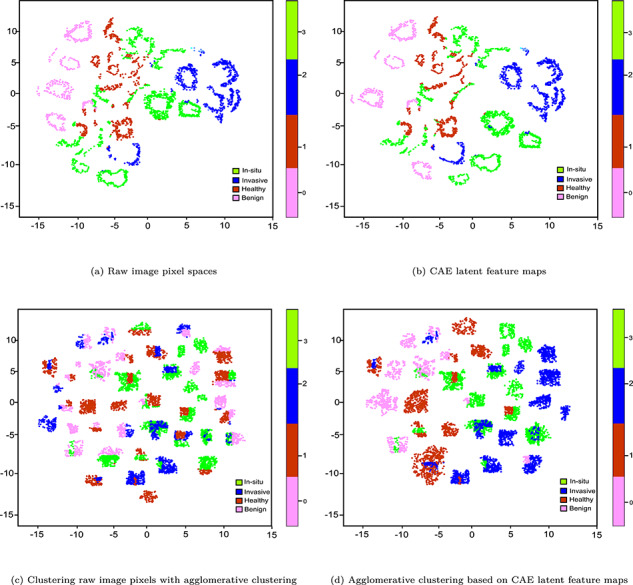
t-SNE plots of different stages in clustering breast microscopy images.

Inspired by Rhee et al. [85], we further qualitatively assess whether the learned representation can express the biological characteristics of the patients. The raw image pixels, encoder’s output (i.e. LF maps), the clusters generated by AC and clustering with AC using LF maps are plotted in Figure 4. From the rightmost sub-figure, we can observe moderately low distinctive patterns between four types of breast cancer subtypes in the t-SNE plot. For example, specific breast cancer subtype has the worst prognosis, e.g. basal, followed by HER2, Luminal B and Luminal A [86]. The reason is that each subtype has distinctive molecular characteristics from other cancer types, which is also reflected in the microscopy images. Although microscopy images of ‘in-situ carcinoma’ and ‘invasive carcinoma’ patients are moderately well-separated, ‘healthy’ and ‘benign’ patients are mostly mixed, albeit not all these patterns are visible in the t-SNE plot with raw images. The final output of AC on CAE-based LF maps is slightly better the best base clustering algorithm (AC, in this case). The latent image features learned by the CAE are better than the raw image pixels, which eventually tends to slightly improve the separability of the microscopy images, albeit not all these patterns are visible in the t-SNE plot of raw microscopy images.

**Table 4:** Clustering result comparison for three bioinformatics use cases

**Use case**	**AE type**	**Clustering algorithm**	**ACC**	**NMI**	**ARI**	**Homogeneity**	**Completeness**
		DBSCAN	0.71	0.70	0.63	0.56	0.65
		OPTICS	0.70	0.69	0.65	0.57	0.67
	AE	Gaussian Mixture	0.65	0.63	0.53	0.52	0.58
		AC	0.72	0.72	0.62	0.68	0.69
		K-means	**0.73**	**0.74**	**0.63**	**0.70**	**0.71**
		DBSCAN	0.78	0.75	0.69	0.67	0.73
		OPTICS	0.80	0.83	0.84	0.72	0.75
	CAE	Gaussian Mixture	0.73	0.71	0.65	0.67	0.68
		AC	**0.85**	**0.83**	**0.84**	**0.75**	**0.77**
Bioimaging		K-means	0.82	0.81	0.80	0.73	0.74
		DBSCAN	**0.75**	**0.76**	**0.70**	**0.65**	**0.73**
		OPTICS	0.72	0.73	0.67	0.58	0.69
	VAE	Gaussian Mixture	0.67	0.69	0.56	0.57	0.65
		AC	0.72	0.72	0.62	0.68	0.69
		K-means	0.71	0.72	0.66	0.69	0.70
		DBSCAN	0.74	0.73	0.68	0.66	0.71
		OPTICS	**0.83**	**0.81**	**0.82**	**0.70**	**0.73**
	LSTM	Gaussian Mixture	0.70	0.69	0.67	0.68	0.69
		AC	0.80	0.82	0.81	0.74	0.75
		K-means	0.81	0.79	0.81	0.75	0.76
		DBSCAN	0.80	0.81	0.80	0.79	0.80
		OPTICS	0.83	0.84	0.85	0.81	0.82
	AE	Gaussian Mixture	0.82	0.83	0.82	0.81	0.82
		AC	0.80	0.74	0.73	0.76	0.77
		K-means	**0.85**	**0.86**	**0.87**	**0.82**	**0.83**
		DBSCAN	0.85	0.86	0.84	0.82	0.83
		OPTICS	**0.87**	**0.86**	**0.88**	**0.84**	**0.85**
	CAE	Gaussian Mixture	0.78	0.79	0.77	0.78	0.79
		AC	0.85	0.84	0.86	0.83	0.82
Text clustering		K-means	0.84	0.85	0.83	0.81	0.81
		DBSCAN	0.82	0.83	0.81	0.80	0.81
		OPTICS	0.84	0.85	0.83	0.82	0.82
	VAE	Gaussian Mixture	0.85	0.86	0.85	0.82	0.83
		AC	0.81	0.79	0.80	0.77	0.78
		K-means	**0.86**	**0.87**	**0.88**	**0.84**	**0.85**
		DBSCAN	0.87	0.88	0.86	0.84	0.85
		OPTICS	**0.91**	**0.92**	**0.93**	**0.90**	**0.89**
	LSTM	Gaussian Mixture	0.82	0.80	0.80	0.81	0.80
		AC	0.88	0.86	0.87	0.84	0.85
		K-means	0.84	0.85	0.86	0.83	0.82
		DBSCAN	**0.82**	**0.83**	**0.81**	**0.79**	**0.80**
		OPTICS	0.81	0.82	0.80	0.77	0.79
	AE	Gaussian Mixture	0.74	0.73	0.73	0.72	0.71
		AC	0.81	0.83	0.84	0.78	0.79
		K-means	0.82	0.83	0.81	0.79	0.76
		DBSCAN	0.84	0.85	0.83	0.79	0.80
		OPTICS	0.83	0.80	0.84	0.78	0.79
	CAE	Gaussian Mixture	0.80	0.76	0.71	0.74	0.76
		AC	**0.86**	**0.85**	**0.87**	**0.81**	**0.83**
Clustering GE		K-means	0.83	0.84	0.82	0.78	0.77
		DBSCAN	**0.84**	**0.82**	**0.85**	**0.82**	**0.81**
		OPTICS	0.80	0.81	0.78	0.76	0.78
	VAE	Gaussian Mixture	0.75	0.76	0.77	0.70	0.69
		AC	0.83	0.81	0.80	0.79	0.80
		K-means	0.81	0.82	0.80	0.78	0.75
		DBSCAN	0.86	0.87	0.88	0.82	0.83
		OPTICS	0.84	0.83	0.82	0.80	0.81
	LSTM	Gaussian Mixture	0.81	0.79	0.77	0.79	0.78
		AC	**0.87**	**0.88**	**0.89**	**0.83**	**0.84**
		K-means	0.80	0.79	0.81	0.76	0.77

The overall best results for a specific use case are highlighted in cyan, while the best results based on other autoencoders are highlighted in bold.

### 3.3 Clustering semantically similar biomedical texts

With the exponential increase of online resources, e.g. scientific articles published, sentiments about drugs, diseases and treatment in the biomedical domain, there is a need to build automated systems to extract hidden knowledge from the unstructured texts [87]. For example, sentiment analysis of drugs can provide valuable insights, help decision-making and improve monitoring public health by revealing collective experiences, particularly in the pharmaceuticals [88]. Since most of the unstructured texts are unlabelled, unsupervised text mining is a viable option, which can facilitate the extraction of vast amounts of knowledge on a given topic and draw meaningful conclusions that are not possible otherwise [87]. We used 215 063 reviews from https://www.drugs.com/ on specific drugs, conditions and a 10-star rating reflecting overall user satisfaction. Reviews contain information about the effectiveness of the drugs and possible side-effects. We apply a DL-based unsupervised clustering to discover similarities among these reviews, and evaluate whether the clusters generated by the network correspond to the overall patient’s satisfaction with applied medications.

Inspired by the literature [88], we derived three-level polarity labels for overall patient satisfaction and drug effectiveness: ratings under and including 4 as negative, between 5 and 7 as neutral and 8 and above as positive. Given drug review texts, we apply a light preprocessing to normalize the texts, reduce the vocabulary size by avoiding colloquial nature and, to some degree, address the sparsity in word-based feature representations. Then we created a word2vec model, which aims to quantify and categorize semantic similarities between linguistic items based on their distributional properties. First, we initialized the word2vec model’s weights using Google news vectors (https://code.google.com/archive/p/word2vec/). Then to exploit the semantic similarities among the tokens, we fine-tuned it using *gensim* (https://radimrehurek.com/gensim/models/word2vec.html) based on the skip-gram method in which each token from the preprocessed input is embedded into a 300-dimensional real-valued vector. The LSTM-AE based on the literature [67] is depicted in Figure 2 has the following structure:

- Input shape: of size $215,063 \times 300$
- Embedding layer: of size $300 \times 300$
- LSTM layer: 128 hidden units
- BN layer: 128 hidden units
- Dropout layer: dropout rate of 20% as weight constraint
- Repeat vector: $300 \times 128$
- LSTM layer: 300 hidden units
- BN layer: 300 hidden units
- Dropout layer: dropout rate of 20% as weight constraint.

During the RL phase, encoder learns fixed-length vector representation of the input texts, while the decoder uses this representation to reconstruct the original vector using the current hidden state and the value predicted at the previous time-step. The probabilistic encoder as an approximation function maps the input into a distribution. Then, the generative probabilistic decoder tries to generate the original sample by means of conditional probability. Next, clustering steps are similar to CAE we discussed before.

The best clustering results are reported in Table 4. In particular, with the best hyperparameters, OPTICS clustering algorithm performs the best based on the LF generated by the LSTM-AE (highlighted in cyan), giving an ARI of 0.85, an NMI of 0.83 and an ACC of 0.84 in which each cluster contains only members of a single class (either negative, positive and neutral) in 75% of the cases and in 77% of the cases all members of a given class are assigned to the same cluster.

**Fig. 5:**
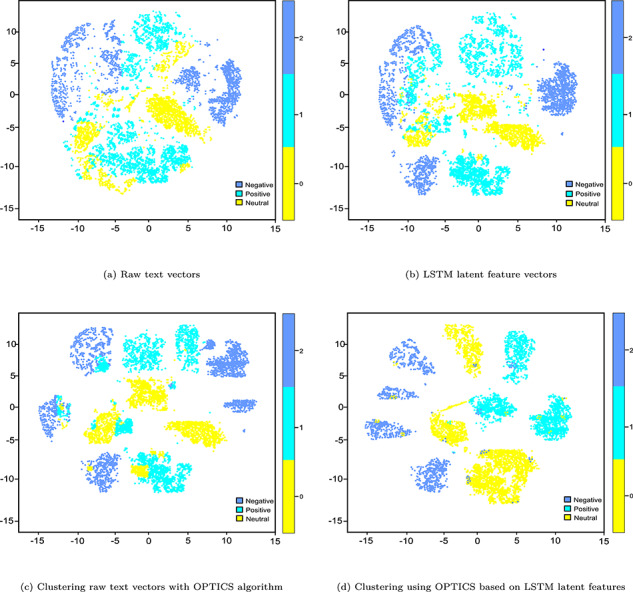
t-SNE plots of different stages in text clustering.

Similar to bioimaging use case, we qualitatively assess whether the learned representations can express characteristics of different types of reviews, raw text vectors, encoder’s output (i.e. LSTM-AE latent vectors), clusters generated by the OPTICS clustering algorithm and clustering with the OPTICS clustering algorithm on LSTM-AE based LF vectors are plotted in Figure 5. From Figure ??, we can observe highly distinctive patterns between three types of reviews in the t-SNE plot. In particular, the final output of OPTICS clustering on LSTM-AE-based LF vectors is moderately better than that of one solely based on the best base clustering algorithm (the OPTICS clustering algorithm, in this case). The reason is that LSTM learned features are better for clustering than the raw review texts. To support this argument, we note that not all these patterns are clearly visible in the t-SNE plot of raw review texts.

Further, since OPTICS is inherently better for sequences, it leads to a slightly better separability of the review texts. In particular, ‘negative’, ‘positive’ and ‘neutral’ reviews are well-separated, even though ‘negative’ and ‘neutral’ reviews are slightly mixed in the t-SNE plots. The reason could be the colloquial nature of review texts in which reviews about drugs were provided a neutral rating but still contain negative words. Consequently, embedding vectors also got ‘contaminated’ with both negative and neutral sentiments.

### 3.4 Clustering GEs

A previous study [10] focused on analyzing GE data using different clustering methods and proximity measures. It reveals that the finite mixture of Gaussians, followed closely by K-means, exhibited the best performance in terms of recovering the true structure of the data. For this experiment, we aim to see how DNN architecture can be used for a similar purpose. The dataset used for this example is a random subset of The Pan-Cancer Analysis Project [89], in which data from thousands of patients with primary tumors occurring in different sites of the body covering 12 tumor types were assembled.

The random extraction contains only RNA-Seq data from 801 patients, each having 20 531 attributes covering breast carcinoma (BRCA), renal kidney carcinoma (KIRC), colon adenocarcinoma (COAD), lung adenocarcinoma (LUAD) and prostate adenocarcinoma (PRAD). Samples are stored row-wise, and attributes of each sample are RNA-Seq GE levels measured by the Illumina HiSeq platform. A dummy name is given to each attribute, but all the attributes are ordered consistently with the original submission. Since GE data are very high dimensional and a significant number of genes have a small or no effect on the tumor, making them very weak features [90], we hypothesize that VAE-based RL can be more effective at learning hierarchical features. Schematic representation of the VAE-based clustering is depicted in Figure 3, which is architecturally a 12-layer VAE in which the BN layer is used before non-linearities and ReLU activation function in every layer. The layer-wise structure of VAE is as follows:

- Input layer: of size $32 \times 1048,576$
- Dense layer: of size $32 \times 256$
- BN layer: of size $32 \times 256$
- Dropout layer: dropout rate of 20% as weight constraint.
- Dense layer: of size $32 \times 32$
- BN layer: of size $32 \times 2$
- Dropout layer: dropout rate of 20% as weight constraint.
- Dense layer: of size $32 \times 2$
- Lambda layer: of size $32 \times 2$
- Dense layer: of size $32 \times 1048,576$
- Dropout layer: dropout rate of 20% as weight constraint.
- Dense layer: of size $32 \times 1048576$.

**Fig. 6:**
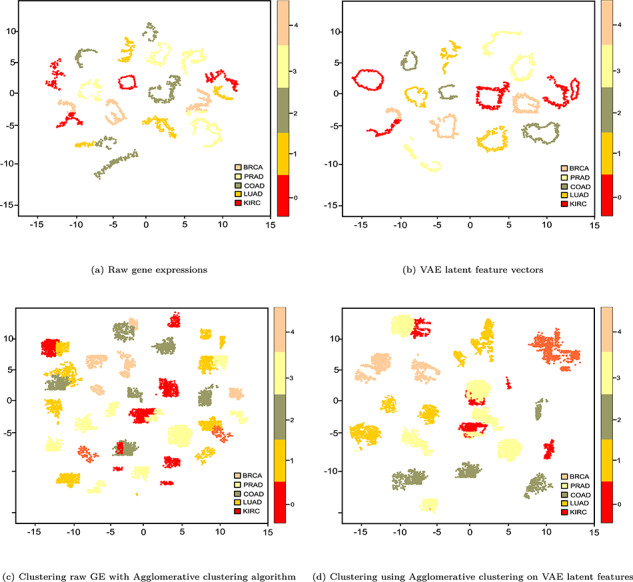
t-SNE plots of different stages of clustering GEs.

During the RL phase, the probabilistic encoder acts as an approximation function to map the input into a distribution. Then, the generative probabilistic decoder tries to generate the original sample by means of conditional probability. Then the clustering steps are similar to CAE we discussed before, whereas clustering results are reported in Table 4 with different metrics in which the AC algorithm performs the best with optimal hyperparameters based on LSTM-AE based LF (highlighted in cyan). In particular, we observed an ARI of 0.87, an NMI of 0.88 and an ACC of 0.89 in which each cluster contains only members of a single class (either BRCA, KIRC, COAD, LUAD and PRAD) in 83% of the cases and in 84% of the cases all members of a given class are assigned to the same cluster. Similar to bioimaging and text clustering, we qualitatively assess whether learned representation can express biological characteristics of patients, raw GE profiles, LF vectors and clusters generated by AC on LF are plotted in Figure 6.

From Figure 6d, we can observe moderately high distinctive patterns between five types of cancer patients in the plot. In particular, BRCA, COAD and LUAD patients are clearly clustered, even though PRAD and KIRC patients are moderately mixed and not separated well. The final output of the AC using CAE-based LF is slightly better than that of one solely based on the best base clustering algorithm (AC algorithm, in this case). The reason is LSTM-AE learned LF that are more quality ones than raw GE data, which eventually tends to a slightly better separability of the GE profiles. As seen in the t-SNE plot, not all these patterns clearly visible in the raw GE profiles.

### 3.5 Discussion and comparative analysis

The clustering results summarized in Table 4 look promising except for the bioimaging use case, albeit CEN performed the best. There could be several reasons for this low confidence in separating the images. One could be the low number of samples (i.e. 400 images). Deep architectures usually require a higher number of samples for generalization, which indicates that these techniques are not suitable for small-sized data sets. In contrast, approach based on LSTM-AE with OPTICS also perform moderately and better than AE and VAE-based approaches, giving an ARI of 0.83, an NMI of 0.81 and an ACC of 0.82, where each cluster contains only members of a single class in 70% of the cases and in 73% of the cases all members of a given class are assigned to the same cluster. The MLP-based AE performs worst, even though K-means performs best among all the base clustering algorithms. For the biomedical texts and GE clustering, LSTM AE-based approach performs best, albeit approach based on CAE with the same base clustering algorithm also performs moderately good.

For text clustering, we experience an ARI of 0.83, an NMI of 0.81 and an ACC of 0.82 in which each cluster contains members of a single class only in 70% of the cases and in 73% of the cases all members of a given class are assigned to the same cluster. Overall, AE performs the worst, even though K-means performs the best among the base clustering algorithms. In contrast, for clustering GE, approach based on CAE with the same base clustering algorithm performed moderately well giving an ARI of 0.86, an NMI of 0.85 and an ACC of 0.87 in which each cluster contains only members of a single class in 81% of the cases and in 83% of the cases all members of a given class are assigned to the same cluster. Here the MLP-based AE performs worst, even though the DBSCAN performs best among all the base clustering algorithms.

In summary, for bioimaging, CAE + AC turns out to be the best option, while CAE + OPTICS also performs well. In contrast, AC and OPTICS performed the best on LF generated by LSTM-AE for both GE and text clustering. To summarize, results covering three different use cases show that clustering results depend on the data type, quality of RL and base clustering algorithms. It turns out that different problems require different techniques, i.e. there is no ‘one size fit all’.

## 4 Conclusions and outlook

In this paper, we provide a comprehensive review of DL-based clustering approaches for bioinformatics research. Clustering results in three different use cases covering different types of data show that approaches based on DL outperform ML-based clustering algorithms. However, overall evaluations are hindered due to the limited amount of labeled data used for the bioimaging and clustering GE. However, neural networks typically require many samples to converge well toward generalization. This study suggests several future directions and an outlook for an improved clustering analysis for bioinformatics use cases:

$\bullet $ Firstly, transfer learning can be employed by means of pretrained models, e.g. Inception, AlexNet, ResNet, DenseNet and VGG16/19 to extract deep features from the images. Weights of the first layers are kept intact, and only the last few layers are fine-tuned to get an improved feature representation. This could then be used to improve the DL-based clustering analysis by reducing the input dimensions to a lower number of features. However, since CNN-based pretrained models are trained on general-purpose images (e.g. ImageNet), they are often not suitable for bioimaging. U-Net [91] can be a better option, which is used for biomedical image segmentation. It consists of a contracting path to capture the context in an asymmetric expanding way that enables precise localization from the biomedical images and outperforms sliding-window based CNN on bioimaging tasks. Further experiments should show whether these improvements also carry over to DL-based clustering.
$\bullet $ Secondly, it would be worth investigating the effect of DSAE [92] on LQ biomedical images and learned representation robust against partial corruption, i.e. partially corrupted inputs should yield almost the same representation. Then, the latent space can be fed to a base clustering algorithm and might learn a better clustering assignment. The unsupervised initialization of layers with a specific denoising criterion would help to capture new structure in the input distribution, which in turn would lead to intermediate representations better suited for subsequent learning tasks such as supervised classification.
$\bullet $ Thirdly, in case of limited labeled data setting, semi-supervised learning could be employed to reduce the need for a large number of labeled examples and instead utilize unlabeled ones. Unsupervised data augmentation [93], for example, achieves state-of-the-art results on a wide variety of language and vision tasks.
$\bullet $ Fourthly, the model ensemble technique could help the networks to achieve improved performance compared to the predictions from single models by reducing the generalization error. It could be achieved by training multiple model snapshots during a single training and by combining their predictions to make an ensemble prediction, i.e. snapshot neural ensemble [94].
$\bullet $ Fifthly, one of the tricky drawbacks of word embedding is that words with multiple meanings are conflated into a single representation in the semantic space. Consequently, polysemy and homonym may occur multiple times. Further, out of vocabulary (OOV) is an issue. For the former, sentence or paragraph embedding approaches or transformer models like BERT [95] (where the encoder reads the entire sequence of words at once that allows the model to learn the context of a word based on its surrounding words) can be used. For the latter issue, using other word embedding methods resilient to OOV can be employed, e.g. fastText.

We believe researchers will find it valuable to apply deep architectures for clustering analysis to advance bioinformatics research. However, many potential challenges remain, including heterogeneous and imbalanced data, interpretation of DL results in an unsupervised setting and selection of appropriate architectures and hyperparameters [28]. In particular, albeit DL-based approaches have shown tremendous success in solving many bioinformatics research problems, they are perceived mostly as ‘black box’ methods because of the lack of understanding of their internal functioning [90]. Further, LF learned by different AE architectures are not easily interpretable, which is a serious drawback. Hence, interpretability is essential to provide insights into what features captured during the RL what attributes of the samples are the clusters based on, e.g. interpretability is a key to generate insights on why and how a certain prediction has been made by the model (e.g. most important biomarkers exhibit shared characteristics while clustering patients having a certain cancer type, which can help in recommending more accurate treatments and drug repositioning). Further, the ‘right to explanation’ of the EU GDPR [96] gives patients similar rights and discuss algorithmic diagnosis decision making and fairness across bioinformatics research scenarios.

In the future, we intend to extend this work by (i) alleviating more samples by combining genomics data from different sources and training a multimodal architecture, (ii) comparing studies on clustering based on feature extracted by DNN vs. PCA and (iii) improving the explanations about the predictions using both ante-hoc and post-hoc approaches. In particular, we plan to employ multimodality [55], since multiple factors are involved in disease diagnosis (e.g. estrogen, progesterone and epidermal growth receptors in breast cancer), AI-based diagnoses might not be trustworthy solely based on a single modality, which demands the requirements of multimodal features (e.g. DNA methylation, GE, miRNA expression and CNVs data) with a reversed time attention model and Bayesian deep learning [97].

## Key points

$\bullet $ DL, based on DNN architectures, has shown huge success in image recognition, speech recognition and natural language processing as well as bioinformatics covering omics, bioimaging, genetics and texts mining.
$\bullet $ DNN architectures such as CAE can learn better feature representations by mapping a high-dimensional input space to a lower-dimensional feature space based on it, clustering assignments can be done using different ML-based clustering algorithms before optimizing the clustering objective iteratively.
$\bullet $ Clustering result itself depends on the type of input data, quality of the RL and base clustering algorithms. This suggests that different problems require different techniques, i.e. there is no ‘one size fit all’.
$\bullet $ The comprehensive review and evaluations of state-of-the-art DL-based clustering analysis approaches provide valuable insights and motivates researchers to extend and apply similar approaches to other emerging bioinformatics research problems, where DL-based approaches outperform classical clustering analysis algorithms.
$\bullet $ Since the representation learned by different AE architectures is not easily understandable, interpretability is required to provide insights into what features and attributes of the samples are captured are clusters based on.
$\bullet $ Python notebooks provided will help bioinformatics researchers to reproduce the result interactively, extend the implementations by changing the network architectures, optimize the hyperparameters and customize for other bioinformatics research problems.

## Conflict of interest

We declare that there is no conflict of interest regarding the publication of this article.

## Abbreviations and acronyms

MC probably best mapping abbreviation to expanded form. We can also use some abbreviation packages here.

$\bullet $ Adversarial Autoencoder (AAE)
$\bullet $ Adjusted Rand Index (ARI)
$\bullet $ Agglomerative Clustering (AC)
$\bullet $ Autoencoders (AEs)
$\bullet $ Autoencoder Loss with Mixture Model for Clusters (ALWMMFC)
$\bullet $ Bayesian Deep Learning (BDL)
$\bullet $ Batch Normalization (BN)
$\bullet $ BreAst Cancer Histology images (BACH)
$\bullet $ Convolutional Neural Network (CNN)
$\bullet $ Centroid-based Clustering (CC)
$\bullet $ Clustering Classification (CC)
$\bullet $ Clustering Assignment Hardening (CAH)
$\bullet $ Clustering Assignment Hardening Loss (CAHL)
$\bullet $ Convolutional Embedded Networks (CEN)
$\bullet $ Convolutional denoising autoencoder (CDAE)
$\bullet $ Clustering and RL with Feature Drift Compensation (UMMC)
$\bullet $ Clustering using CNN (CCNN)
$\bullet $ Convolutional Autoencoder (CAE)
$\bullet $ Clustering Accuracy (ACC)
$\bullet $ Clustering Quality (CQ)
$\bullet $ Deep Learning (DL)
$\bullet $ Deep Neural Networks (DNN)
$\bullet $ Deep Belief Networks (DBN)
$\bullet $ Deep Embedded Clustering (DEC)
$\bullet $ Deep Clustering Network (DCN)
$\bullet $ Dimensionality Reduction (DR)
$\bullet $ Distribution-based Clustering (DC)
$\bullet $ Density-based Clustering (DC1)
$\bullet $ Deep Continuous Clustering (DCC)
$\bullet $ Discriminatively Boosted Clustering (DBC)
$\bullet $ Denoising Stacked AE (DSAE)
$\bullet $ Deep Embedding Network (DEN)
$\bullet $ Deep Clustering with CAE embedding (DEPICT)
$\bullet $ Deep Adaptive Image Clustering (DAC)
$\bullet $ Deep Learning with Non-parametric Clustering (NMMC)
$\bullet $ Expectation Minimization (EM)
$\bullet $ Gaussian Mixture Model (GMM)
$\bullet $ Generative Adversarial Networks (GAN)
$\bullet $ Gene Expressions (GEs)
$\bullet $ Greedy Layer-wise (GLW)
$\bullet $ Hierarchical Clustering (HC)
$\bullet $ High Quality (MQ)
$\bullet $ Info Maximization and Self-augmentation Loss (IMSAL)
$\bullet $ Joint Unsupervised Learning of Representation for Images (JULE)
$\bullet $ Kullback-Leibler Divergence (KLD)
$\bullet $ Latent Features (LFs)
$\bullet $ Learning Rate (LR)
$\bullet $ Locality preserving loss (LPL)
$\bullet $ Locality Preserving and Group Sparsity (LP+GS)
$\bullet $ Low Quality (LQ)
$\bullet $ Learning Latent Representations for Clustering (IMSAT)
$\bullet $ Long Short-Term Memory (LSTM)
$\bullet $ Machine Learning (ML)
$\bullet $ Multilayer Perceptron (MLP)
$\bullet $ Max Margin for Mixture Components (MMMC)
$\bullet $ Medium Quality (MQ)
$\bullet $ Neural Network (NN)
$\bullet $ NN-based Clustering using Pairwise Constraints Clustering (NNCPC)
$\bullet $ Normalized Mutual Information (NMI)
$\bullet $ Pairwise Constraints (PC)
$\bullet $ Partitioning Around Medoids (K-medoids)
$\bullet $ Principal Component Analysis (PCA)
$\bullet $ Permutation Model (PM)
$\bullet $ Representation Learning (RL)
$\bullet $ Reconstruction Loss (RL1)
$\bullet $ Self-Organizing Maps (SOM)
$\bullet $ Stacked Convolutional Autoencoder (SCAE)
$\bullet $ Stochastic Gradient Descent (SGD)
$\bullet $ Transfer Learning (TL)
$\bullet $ Unsupervised Data Augmentation (UDA)
$\bullet $ Variational Autoencoder(VAEs)
$\bullet $ Within-cluster Sum of Squares (WCSS).

## Notes

**Md. Rezaul Karim** is a Researcher at Fraunhofer FIT, Germany and a Ph.D. candidate at RWTH Aachen University, Germany. He is working towards developing a distributed knowledge pipeline with knowledge graphs and neural networks towards making them explainable and interpretable. His research interests include machine learning, knowledge graphs, bioinformatics, and explainable artificial intelligence (XAI).

**Oya Beyan** is a Research Fellow at RWTH Aachen University. She received BSc, MSc, and Ph.D. degrees from the Middle East Technical University, Ankara, Turkey. Between 2012 and 2015, she was the research fellow, adjunct lecturer, and the Head of Innovative Personalised Medicine Technologies Research Centre at NUI Galway, Ireland. Her research interests include biomedicine, bioinformatics, and data management.

**Achille Zappa** is a Postdoctoral Researcher at Insight Centre for Data Analytics and Data Science Institute at NUI Galway, Ireland. His research interests include Semantic Web, semantic data mashup, bioinformatics, data management, and knowledge engineering. He received his Ph.D. in bioengineering from the University of Genoa, Genoa, Italy.

**Ivan G. Costa** received his Ph.D. in computational biology from the Max Planck Institute for Molecular Genetics, Berlin, Germany. Currently, he is a Professor and Head of Institute of Computational Genomics, RWTH Aachen University Hospital, Germany. His research interests include computational biology, molecular genetics, and machine learning.

**Dietrich Rebholz-Schuhmann** is a Professor of medicine and Director of German National Library of Medicine, University of Cologne, Germany. He was the director of Insight Centre for Data Analytics, and a professor of informatics at NUI Galway, Ireland, group leader at European Bioinformatics Institute, and an oberassistent PI at University of Zurich, Switzerland. His research interests include Semantic Web & linked data, biomedical texts mining, and bioinformatics.

**Michael Cochez** is an Assistant Professor at VU Amsterdam, the Netherlands. Before his postdoc at Fraunhofer FIT, he received his Ph.D. and M.Sc. degrees in Mathematical Information Technology from the University of Jyväskylä, Finland, and his B.Sc. degree in Information Technology from the University of Antwerp, Belgium. His research interests include data analysis and knowledge representation.

**Stefan Decker** is the director of Fraunhofer FIT and the Professor of Computer Science at RWTH Aachen University. He was the director of Insight Centre for Data Analytics, and professor of informatics at NUI Galway, Ireland between 2006 and 2015. His research interests include Semantic Web & linked data, knowledge representation, and neural networks.
